# A Comparison of the Elastic Properties of Graphene- and Fullerene-Reinforced Polymer Composites: The Role of Filler Morphology and Size

**DOI:** 10.1038/srep31735

**Published:** 2016-08-22

**Authors:** Chang-Tsan Lu, Asanka Weerasinghe, Dimitrios Maroudas, Ashwin Ramasubramaniam

**Affiliations:** 1 Department of Chemical Engineering, University of Massachusetts, Amherst, MA 01003, USA; 2 Department of Physics, University of Massachusetts, Amherst, MA 01003, USA; 3 Department of Mechanical and Industrial Engineering, University of Massachusetts, Amherst, MA 01003, USA

## Abstract

Nanoscale carbon-based fillers are known to significantly alter the mechanical and electrical properties of polymers even at relatively low loadings. We report results from extensive molecular-dynamics simulations of mechanical testing of model polymer (high-density polyethylene) nanocomposites reinforced by nanocarbon fillers consisting of graphene flakes and fullerenes. By systematically varying filler concentration, morphology, and size, we identify clear trends in composite stiffness with reinforcement. To within statistical error, spherical fullerenes provide a nearly size-independent level of reinforcement. In contrast, two-dimensional graphene flakes induce a strongly size-dependent response: we find that flakes with radii in the 2–4 nm range provide appreciable enhancement in stiffness, which scales linearly with flake radius. Thus, with flakes approaching typical experimental sizes (~0.1–1 μm), we expect graphene fillers to provide substantial reinforcement, which also is much greater than what could be achieved with fullerene fillers. We identify the atomic-scale features responsible for this size- and morphology-dependent response, notably, ordering and densification of polymer chains at the filler–matrix interface, thereby providing insights into avenues for further control and enhancement of the mechanical properties of polymer nanocomposites.

Graphene—a planar sheet of *sp*^2^-bonded carbon atoms—has been the subject of significant experimental and theoretical investigation over the last decade both from a materials engineering perspective, due to its exceptional mechanical and electronic properties, as well as from a fundamental physics perspective, due to a host of exotic properties as a low-dimensional material^1^,^2^,^3^. Among several potential applications, the use of graphene flakes as nanoscale fillers in polymer-matrix composites appears to be particularly promising with several reports of significant impact on the polymer’s properties at relatively low graphene loading, including improvement in stiffness and strength, enhancement in electrical and thermal conductivity, elevation of the glass transition temperature, and reduction in gas permeability^4^,^5^,^6^,^7^. While some of these properties can be realized with other silicate or nanocarbon (e.g., nanotubes or fullerenes) fillers, graphene-based fillers offer a broad combination of these properties with the added benefits of relative ease of synthesis and dispersion along with low cost^4^,^8^. With sustained improvements in synthesis, exfoliation, and functionalization techniques, graphene-based fillers are, thus, poised to enable the development of new polymer composites with unique functionality and novel applications.

From a computational perspective, toward a fundamental understanding of filler effects on polymer-matrix composite properties and function, the role of nanocarbon fillers has been studied in much detail focusing on the role of the filler on the structure, dynamics, and mechanical response of polymer nanocomposites^9^,^10^,^11^,^12^,^13^,^14^,^15^,^16^,^17^,^18^,^19^. Molecular-dynamics (MD) simulations have proved extremely useful in providing fundamental insights into the nature of the filler–matrix interface, which is of particular significance for high-surface-area fillers such as graphene. Due to the high surface-to-volume ratio of graphene, polymer chains tend to stretch and pack along the graphene filler surface leading to higher densification in the vicinity of the filler relative to the bulk, as reported by Li *et al*.^9^ and Harmandaris and coworkers^10^,^11^,^12^. These studies also show that the formation of densely packed regions at the graphene–matrix interface leads to slower relaxation dynamics for the polymer chains. While such studies have focused primarily on understanding the organization and dynamics of polymer chains at graphene surfaces, detailed systematic computational studies of the mechanical behavior of graphene–polymer composites are still lacking. A recent study^16^ has examined the mechanical properties of functionalized graphene (graphene oxide) paper infiltrated with polymer; that work, however, probed the limit opposite to the regime of typical interest, namely, that of low filler loading.

The purpose of this paper is to conduct a detailed analysis of the elastic response and properties of graphene–polymer composites at low-to-moderate graphene loading and to identify the underlying mechanisms responsible for graphene filler-induced reinforcement. Using MD simulations, we study the mechanical response of a model polymer (chosen for simplicity as high-density polyethylene; HDPE), reinforced by graphene flakes. The choice of HDPE as the polymer matrix is simply one of convenience that allows us to focus on the essential physics of matrix–filler interactions without additional complications arising from the matrix itself. To understand the importance of polymer chain packing at the filler surface in controlling the mechanical response, we also study the mechanical response of polymer-matrix composites with chemically comparable but morphologically distinct fullerene fillers. By systematically varying filler concentration, morphology, and size, as well as inter-chain and chain-filler interactions, we identify certain clear trends in composite stiffness with reinforcement. We have undertaken an extensive statistical sampling over atomic configurations and trajectories relative to the parameters noted above, which establishes clear confidence levels for the observed material response. In particular, we find that, to within statistical error, spherical fullerenes provide a nearly size-independent level of reinforcement of the matrix. In contrast, composites with graphene fillers exhibit reinforcement that depends strongly on the graphene filler size: although sub-nanometer graphene flakes are found to be ineffective fillers, flake sizes in the 2–4 nm range lead to appreciable reinforcement, which scales linearly with flake size, with graphene flakes outperforming with increasing size all the fullerenes examined. We elucidate the fundamental mechanisms responsible for this size- and morphology-dependent response, thereby providing insights into processing strategies for further control and enhancement of the mechanical properties of nanocarbon-reinforced polymers.

## Computational Methods

### Interaction Parameters for Composites with Polyethylene Matrix and Carbon Fillers

The interatomic potentials for both the HDPE matrix and carbon fillers are based on a united-atom model, which treats individual hydrocarbon sub-units (CH_2_ and CH_3_ groups) as a single entity. Such united-atom models are commonly described using the Dreiding potential^20^, which accounts for both bonded and non-bonded interactions. The bonding energy contribution consists of three components, *E*_*bond*_, *E*_*angle*_, and *E*_*dihedral*_, arising from changes in the bond length (*r*), bond angle (*θ*), and dihedral angle (*ϕ*), respectively, which are given by the expressions


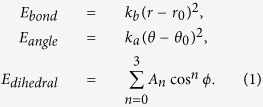


Non-bonded interactions are assumed to follow a standard 12-6 Lennard-Jones form,


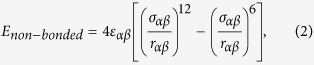


where *α* and *β* denote the two interacting united atoms; atoms within a distance equal to four C-C bond lengths of each other in the same polymer chain do not participate in such non-bonded interactions. Various Dreiding potentials have been used extensively for studies of neat polyethylene (PE)^21^,^22^,^23^ as well as PE composites^9^,^10^,^11^,^12^,^13^,^14^,^15^. Here, we adopt two such parameterizations developed by Buell *et al*.^22^ and Capaldi *et al*.^21^. The relevant parameters in Equations (1) and (2) for these two potentials are listed in Table S1 in the Supplementary Information. While the parameters for the bonded interactions are different for these two potentials, the key difference between the two parameterizations arises from the non-bonded interactions. Specifically, the CH_2_-CH_2_ non-bonded interaction for the potential by Capaldi *et al*. is about 20% stronger than that for the potential by Buell *et al*.; since CH_2_ groups are the dominant component of the polymer matrix, this difference in non-bonded interaction strength results in a stiffer response of the matrix according to the former potential relative to that according to the latter as shown subsequently.

Nanocarbon fillers also are described using a Dreiding potential^24^ whose parameters are listed in Table S2 in the Supplementary Information^24^. Note that in contrast to some studies that have treated fillers (notably fullerenes) as rigid inclusions^14^,^15^, in this study, we allow for fully elastic, deformable fillers. Filler–matrix interactions are described as non-bonded interactions using standard Lorentz-Berthelot mixing rules^25^ resulting in the interaction parameters listed in Table S3 in the Supplementary Information^24^. This is a convenient approximation that allows us to identify fundamental scaling behavior in the composite system with minimal complexity; covalent matrix–filler bonding (through, e.g., functionalized fillers) is beyond the scope of this study and will be reported elsewhere. We note that the mixing rules employed here are by no means unique and other choices are equally if not more valid^26^; nevertheless, we expect that the qualitative details of the structure of the polymer–matrix interface as well as scaling behavior in the mechanical response of the composite, discussed later in this paper, are unaffected by such choices.

Henceforth, for convenience, we will refer to the parameterization by Buell *et al*. as HDPE1 and to that by Capaldi *et al*. as HDPE2; the corresponding parameterizations for the interactions in the composite systems will be referred to as HDPE1/C and HDPE2/C.

### Sample Preparation

Computational models of material samples were prepared with the aim of gathering extensive statistics and identifying clear trends in the elastic response as a function of filler size, concentration, and geometry. The polyethylene matrix was modeled using unbranched CH_3_-(CH_2_)_*n*_-CH_3_ (*n* = 98; “100-mer”) chains. The carbon fillers used in our study are “zero-dimensional”, cage-like fullerenes and two-dimensional (2D), planar graphene flakes. We considered fullerenes of three different sizes, C_60_, C_180_, and C_540_, and graphene flakes of five different sizes, C_61_, C_181_, C_541_, C_1087_, and C_2161_; the three smallest graphene flakes were chosen to closely mimic the carbon content of the three fullerenes examined, thereby facilitating a direct comparison of the role of filler morphology in the composite’s elastic response. The fullerene structures were prepared using the routines by Schwerdtfeger *et al*.^27^, while the graphene flakes were prepared as circular discs.

We used a self-avoiding-random-walk (SAW) algorithm to generate supercell models of random polymer matrices. The SAW step size was chosen as the equilibrium C-C bond length of the polymer backbone (*r*_0_) with changes in orientation at each step being equal to the equilibrium bond angle (*θ*_0_); no two non-bonded atoms were allowed to approach within a distance of one bond length of each other in the SAW. For preparing composite system supercells, we first dispersed fillers in the supercell and thereafter inserted the polymer matrix using a SAW scheme under additional constraints that prohibited collisions between the polymer chains and fillers. Table S4 in the Supplementary Information^24^ lists the various filler–matrix combinations used in this work; ten samples corresponding to each of these combinations were used in the mechanical tests that we conducted to generate reliable statistics.

To ensure reasonably fast convergence of the SAW algorithm, samples were produced at a low initial density (0.5 g/cm^3^) and, hence, required proper thermal equilibration and relaxation. To this end, we first carried out a conjugate-gradient energy minimization of the initial configuration with a force tolerance of 0.01 eV/Å implemented in the MD simulation software LAMMPS^28^. Thereafter, the system was thermalized with isothermal-isobaric (*NPT*) dynamics, implemented in LAMMPS, at 500 K and equilibrated over 100 picoseconds (with a time step of 1 femtosecond) to a pressure of 1 atm using a Nosé-Hoover thermostat and barostat^29^. These times were chosen after extensive testing to ensure minimal oscillations in temperature and pressure by the end of the relaxation cycle. Subsequently, using (*NPT*) dynamics, the samples were quenched by 25 K every 100 ps. Figure S1 in the Supplementary Information displays typical density versus temperature curves for neat as well as nanocarbon-reinforced HDPE. From these calibration curves, one can estimate the glass transition temperature (*T*_*g*_) as indicated by the example constructions in Fig. S1. For neat HDPE—considering both parameterizations of Table S1—we estimated *T*_*g*_ to lie over the range 240–265 K, which contains the typical experimentally reported value of 250 K^30^; the addition of fillers results in a slight elevation of *T*_*g*_ to the range of 260–295 K. The root mean square (RMS) displacement of the polymer united atoms computed over the (*NPT*) annealing schedule exceeds the radius of gyration of the polymer chains in our computational samples, which confirms proper initial equilibration of our samples prior to mechanical testing^31^. For longer polymer chains, the time scales for conformational changes are typically much longer than those that can be accessed with MD and alternate approaches such as connectivity-altering Monte Carlo models^32^ must be adopted for proper equilibration.

### Mechanical Testing Procedure

The mechanical response of polymers is extremely sensitive to temperature. Here, we chose to carry out all of our tests at a temperature of 150 K such that all of our samples were well within the glassy regime (see Fig. S1). The choice of test temperature and interatomic potentials simply establishes a canonical glassy polymer model, which is useful for elucidating trends in the elastic response of the composite rather than predicting quantitatively precise values. Each randomly prepared and equilibrated (to 150 K) specimen was subjected to independent uniaxial tensile tests along each of the three Cartesian directions using Nosé-Hoover (*NPT*) dynamics. Fixed strain increments were applied to a chosen simulation supercell vector at intervals of 10 ps to result in an overall strain rate of 10^9^/s, while the remaining cell vectors were relaxed to attain a pressure of 1 atm. The virial stress (*σ*_*ij*_), defined as


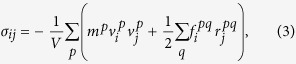


was calculated by averaging over the last 5 ps of every applied strain increment. In Eq. (3), *V* is the volume of the simulation cell; *m*^*p*^ and 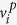 are the mass and *i*-th component of the velocity of particle *p*; 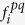 and 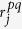 are the *i*-th component of the force and the *j*-th component of the separation, respectively, between particles *p* and *q*; with the summations being over all particles in the simulation cell.

## Results and Discussion

**Figure 1:**
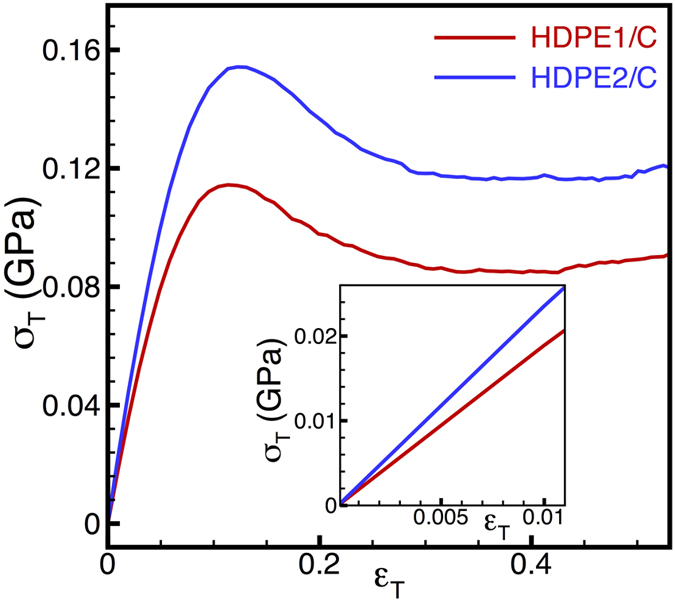
Typical true stress vs. true strain curves from uniaxial tensile straining tests for neat HDPE using HDPE1/C (red lines) and HDPE2/C (blue lines) parameter sets. The inset focuses on the low-strain mechanical response, indicating that the HDPE2/C parameter set predicts a stiffer response than that of the HDPE1/C parameter set.

First, we consider the mechanical response of neat HDPE to establish the baseline properties of the matrix prior to insertion of fillers. Figure 1 shows typical stress-strain curves from uniaxial tensile tests on HDPE as a function of interatomic potential. As noted previously, the non-bonded CH_2_-CH_2_ interactions for HDPE2 are about 20% stronger than those for HDPE1, which accounts for the observed stiffer response in the former case. Specifically, at the chosen temperature of 150 K (below *T*_*g*_), the calculated Young’s modulus for the neat polymer is 1.62 ± 0.02 GPa with HDPE1 parameters and 2.02 ± 0.03 GPa with HDPE2 parameters. These values are typical of Young’s moduli of glassy amorphous polymers^33^. As we examine the elastic response of the nanocarbon-reinforced polymer below, we will consistently present results normalized by the elastic properties of the neat polymer to allow for a more representative comparison.

**Figure 2:**
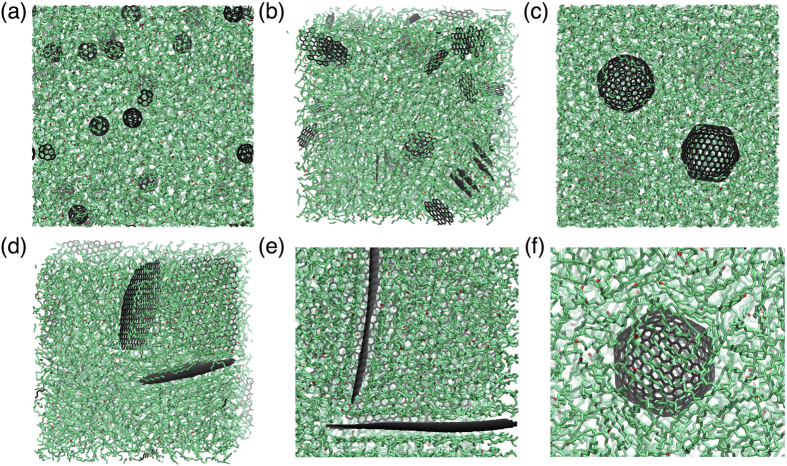
Examples of simulation supercells showing nanocarbon–HDPE composites with nanocarbon fillers consisting of (**a**) C60 fullerenes, (**b**) C61 graphene flakes, (**c**) C540 fullerenes, and (**d**) C541 graphene flakes. The polymer chains are represented by sequences of linked green segments. Magnified views in the vicinity of (**e**) two C1087 graphene flakes showing densification and layering of polymer chains near the matrix–filler interface and (**f**) a C540 fullerene showing similar densification, albeit significantly reduced compared to that of (**e**).

**Figure 3:**
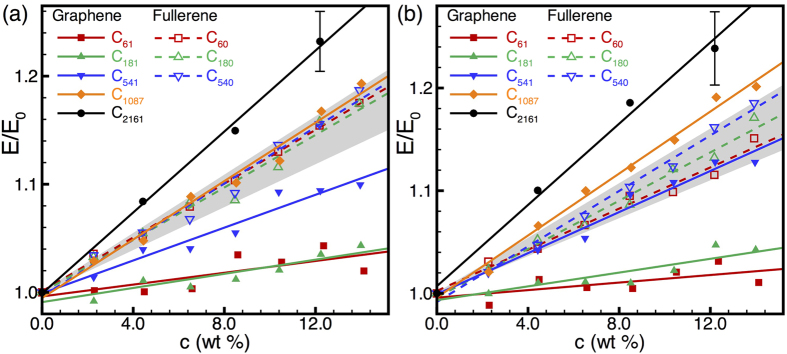
Normalized elastic modulus, E/E_0_, of nanocarbon-HDPE composites as a function of filler concentration, c, for (**a**) HDPE1/C and (**b**) HDPE2/C parameters. The composite modulus (E) is normalized by the corresponding neat HDPE modulus (E_0_) in each case. Filled symbols and solid lines indicate graphene-flake fillers, while open symbols and dashed lines indicate fullerene fillers. Straight lines indicate linear fits to the data; each data point corresponds to a statistical average of 30 tensile straining tests but only one typical error bar is shown in each plot to retain clarity. In each case, the shaded region indicates the rather narrow range of moduli spanned by fullerene-reinforced composites. In contrast, graphene reinforcement shows significant filler-size-dependent stiffening.

Next, we analyze systematically the influence of filler size, morphology, and concentration on the response of nanocarbon-reinforced HDPE. Figure 2 illustrates typical atomic configurations of fullerene- and graphene-reinforced HDPE. In general, the equilibration process results in composites with well dispersed filler. It is only at fairly high concentrations and at small filler sizes that we begin to observe aggregation of fillers, a few examples of which are seen in Fig. 2(a,b) at concentrations of 8.47 and 8.60 wt%, respectively. Interestingly, we find that even with the formation of such aggregated fillers, the fullerenes are always intermixed with HDPE chains, whereas the graphene sheets can spontaneously stack as few-layer graphitic flakes. In practice, depending upon processing conditions, one would also expect aggregation of larger nanoscale fillers. However, the degree of conformational changes required to achieve such aggregation require equilibration times that are well beyond those achievable with standard MD simulations. Thus, our model composites with larger fillers might display a higher degree of dispersion leading to better reinforcement than is to be expected in practice and, consequently, the predicted mechanical properties may be interpreted as upper bounds on the actual response. We carried out extensive statistical simulations of the elastic response of HDPE- nanocarbon composites considering ten tests on large sets of random samples as outlined in Table S4, and the resulting elastic response is summarized in Fig. 3. As expected, with increasing filler concentration, we find a monotonic increase in the elastic modulus of the composite; this increase in modulus with filler concentration is fairly linear. While there is some dependence of the composite response on the non-bonded interaction strength (examined by the two different interaction potentials), these factors only exert a small effect on the overall trends in the composites’ elastic response. The most noteworthy differences between the two potentials, as seen from Fig. 3, are a more noticeable effect of fullerene size on composite stiffness as well as a more rapid increase in composite stiffness with increasing graphene flake size when using HDPE2 parameters. However, it is immediately evident that the response of the composite is extremely sensitive to the morphology of the filler. For small graphene flake sizes (C_61_ and C_181_), the role of the filler is, to within statistical error, inconsequential; it is not until the graphene flake size increases to C_541_ that we begin to see an appreciable improvement in the composite modulus with filler concentration. On the contrary, fullerenes act as effective fillers even at small sizes (e.g., C_60_ and C_180_) at enhancing the composite’s modulus. However, the filler-size effect for fullerenes appears to be much less significant than that for graphene flakes, with results for C_60_, C_180_, and C_540_ spanning a rather narrow band of moduli (with appreciable overlap of error bars) as indicated by the grey shaded regions in Fig. 3. In contrast, we observe a consistent enhancement in the composite modulus with increasing graphene flake size: C_541_ flakes begin to result in comparable modulus enhancement as that of the fullerene fillers, while C_1087_ and C_2161_ flakes outperform the largest fullerenes considered here.

**Figure 4:**
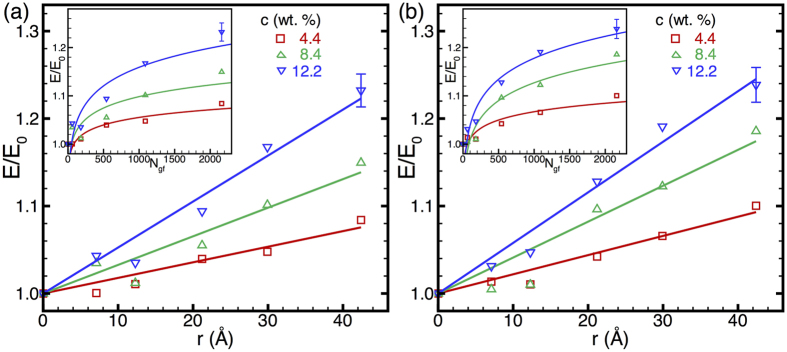
Normalized elastic modulus, *E*/*E*_0_, as a function of graphene flake radius, *r*, for (**a**) HDPE1/C and (**b**) HDPE2/C parameters. Straight lines represent linear fits to the data. In the insets, the normalized moduli are plotted as a function of the number of atoms, *N_gf_*, in each flake of graphene filler (or, equivalently, circular flake area); lines in the insets represent ½-power-law fits to the data. Each data point is a statistical average over 30 tensile straining tests but only a couple of typical error bars are shown in each plot to retain clarity.

The size-dependent stiffening of graphene-reinforced HDPE is elucidated further in Fig. 4, where the composite modulus is plotted as a function of filler size for selected filler concentrations. As seen in Fig. 4, for a given concentration of graphene flakes, we find a nearly linear increase in the composite modulus with the flake radius *r* or, equivalently, as depicted in the insets in Fig. 4, a square-root power-law growth with the number of atoms, *N*_*gf*_, in the flake. This *E*/*E*_0_ ~ *r* or *E*/*E*_0_ ~ *N*_*gf*_^1/2^ at given filler concentration is an important scaling relation for the elastic response of the HDPE-graphene composites. We note that this scaling relation is as yet based only on a limited set of flake sizes in the 1–4 nm range; larger-scale MD simulations with flakes in the 10–100 nm range will be reported in a future publication to verify the validity of this scaling relation over a couple of orders of magnitude of graphene filler size. In any case, the results here are very promising as they establish a clear trend for the enhancement of polymer–graphene composite stiffness, as experimentally relevant graphene flakes have sizes that are typically in the sub-micron range. On the contrary, the limited scope for modulus enhancement with increasing fullerene filler size that is evident from our results—in conjunction with the fundamental challenges associated with the synthesis of such large fullerenes—is indicative of their reduced utility as fillers compared to graphene flakes.

The main computational findings thus far, namely, the linear scaling of composite stiffness with graphene concentration as well as the linear dependence of composite stiffness on the lateral dimensions of the filler are generally consistent with classical composite theory^34^. The stiffness enhancement with increasing concentration of graphene flakes follows from a straightforward mixing-rule argument. In addition, the strong dependence of composite stiffness on graphene flake size can be understood on the basis of continuum shear-lag theory^35^. Being 2D fillers, graphene flakes rely on interfacial shear forces for matrix–filler load transfer. As is well known from shear-lag theory, the reinforcement effect becomes more pronounced with increasing filler aspect ratios (corresponding to increasing flake radii here) as the tensile stress in the filler can build up to the optimal load-carrying capacity of the filler. In contrast, low-aspect-ratio fillers carry a much lower tensile stress than their actual capacity and are, thus, less effective at composite reinforcement. Based on a shear-lag analysis, as applied to recent experiments on polymer–graphene composites^36^, an order-of-magnitude estimate for a critical graphene flake radius for interfacial stress transfer would be in the micrometer range. Such size effects are less relevant for fullerenes, which simply function as stiff isotropic inclusions within a compliant matrix.

**Figure 5:**
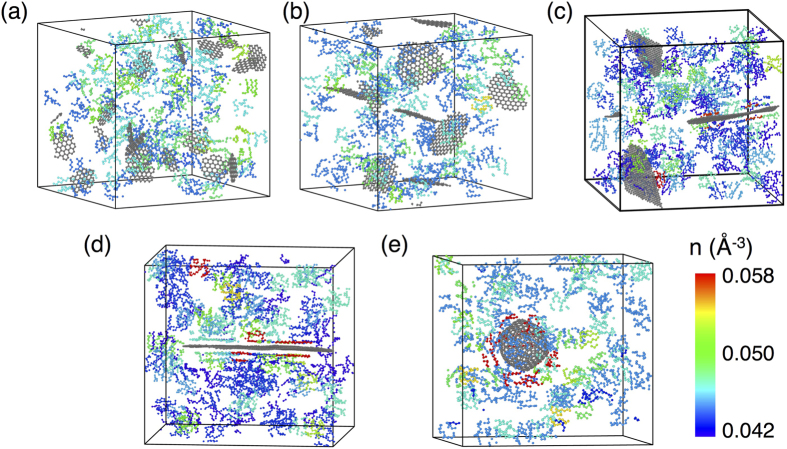
Number density maps of nanocarbon-HDPE composites with fillers consisting of (**a**) C_61_, (**b**) C_181_, (**c**) C_1087_, and (**d**) C_2161_ graphene flakes, as well as (**e**) C_540_ fullerenes. A lower cutoff is imposed on the number density to eliminate uninteresting portions of the polymer matrix and highlight only the higher-density regions. As seen in the maps, larger flakes induce increased ordering of polymer chains at the filler–matrix interface as well as higher local densification of the polymer chains in these regions. Similar but reduced local densification also is evident at the C_540_–matrix interface.

While continuum theory provides a broad understanding of the composite response, there are additional microscopic aspects of the matrix–filler interface, which may become important for load transfer at the nanoscale. For example, consider the case of small graphene flakes (C_61_ and C_181_) dispersed in HDPE: Fig. 5(a,b) depict the density distribution of the polymer matrix for these two composites, which is relatively uniform throughout the simulation cell thus indicating that the filler has little to no influence on the matrix density. On the contrary, when larger C_1087_ and C_2161_ flakes are dispersed in the HDPE matrix [Fig. 5(c,d)], we observe that the polymer chains are densely packed in the vicinity of the filler. Consequently, larger graphene flakes induce layering and densification of the polymer chains in the vicinity of the filler, consistent with the findings of Refs. 9, 10, 11, 12. This layering and densification of polymer chains also can be seen clearly from the atomic configuration of the composite in the vicinity of a C_1087_ flake [Fig. 2(e)]. For large C_540_ fullerenes, whose surfaces tend to undergo faceting and deviation from strictly spherical geometry, we also observe some layering and densification of the matrix in the vicinity of the filler’s facets [Figs 2(f) and 5(e)], which likely explains the slight stiffness enhancement observed in Fig. 3 for this large fullerene relative to smaller C_60_ and C_180_ fullerenes. Nevertheless, it is clear that a graphene flake provides a much larger planar area relative to a faceted fullerene of comparable carbon content for adsorption and subsequent densification of polymer chains. However, due to their low surface area, small graphene flakes cannot induce such a local ordering and densification of the polymer chains.

**Figure 6:**
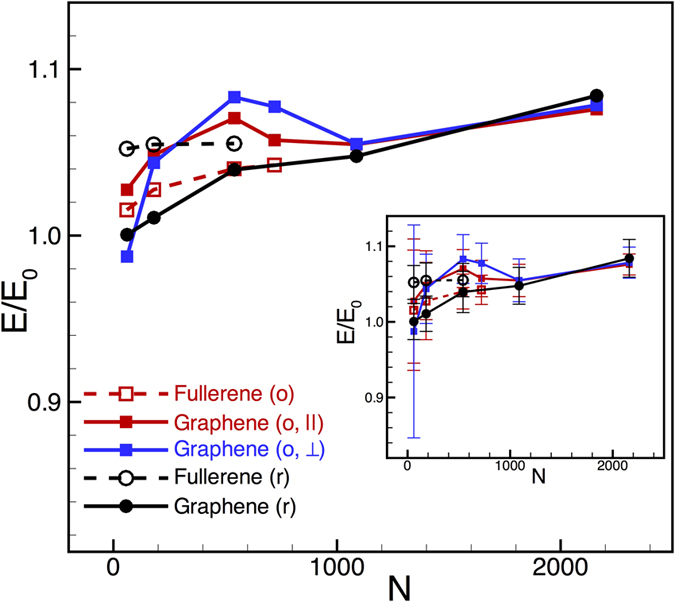
Normalized composite modulus as a function of filler size (expressed in terms of number of carbon atoms, *N*) for fullerenes and graphene flakes of various sizes. The distribution of fillers is either ordered (“o”) (i.e., one filler per simulation cell, which, under periodic boundary conditions, leads to an ordered composite) or random (“r”). For the ordered grapheme flakes, two separate directions of loading, namely, parallel (||) and perpendicular (⊥) to the plane of the flakes, also are considered. The inset displays the same data as shown in the main plot with statistical error bars included. In all cases, the filler concentration is fixed at 4 wt%. The HDPE1/C set of parameters was used in these studies.

To probe further the role of the matrix–filler interface for load transfer, we carried out an additional series of calculations wherein we inserted exactly one filler per simulation cell and calculated the composite modulus for fullerenes and graphene flakes of varying sizes keeping a constant filler-to-polymer weight ratio of 4%. This idealized geometry (denoted as “o” – ordered, in Fig. 6) eliminates complications arising from orientational anisotropy and/or clustering of fillers in the random composites (denoted “r” – random, in Fig. 6). For spherical fullerenes, isotropy implies the same nominal response along all loading direction. For graphene flakes, however, one would expect different responses for loading parallel and perpendicular to the flake; load transfer along the flake ought to be governed by interfacial shear stresses, whereas load transfer normal to the flake would depend upon the strength of adhesion between the flake and polymer. Thus, by simulating the mechanical response parallel and normal to the graphene flakes, one may decouple shear-lag effects from normal load transfer.

Figure 6 displays the results of our calculations for fullerenes and graphene flakes of various sizes. First, for small filler sizes (C_60_ and C_180_ fullerenes; C_61_ and C_181_ graphene), we see that the effect of randomness versus ordering is insignificant, to within statistical error; with larger fillers, the responses are more intelligible although there is still some overlap of error bars. For ordered graphene flakes, we see that the response for loading along the flakes (“o, ||” in Fig. 6) is stiffer than (or at least equal to) that for the randomly oriented flakes, which is to be expected as the ordered flakes are optimally oriented for load-transfer via shear. Interestingly though, for loading normal to the flakes (“o, ⊥” in Fig. 6), the response is just as stiff as that along the flake. Clearly, stiffening in the direction normal to the flake cannot be attributed to a classical shear-lag mechanism. Thus, we conclude that local details of the matrix–filler interface, which ultimately control adhesion, are indeed important, at least for sub-nanometer graphene flakes considered in this study. With increasing graphene flake sizes, one could expect a crossover to a regime where classical shear-lag effects dominant the stiffness response although it remains to be seen if this length-scale can be demarcated with fully-atomistic simulations. In closing, we note that it is not possible to draw from these simulations a direct correlation between the higher degree of polymer densification at the matrix–filler interface and the improved stiffness response for larger flakes; nevertheless, it appears plausible that the layering and densification could improve adhesion between the filler and the matrix and transfer load more efficiently.

## Conclusions

In summary, we have conducted an extensive molecular-dynamics parametric study of the elastic properties of fullerene- and graphene-reinforced HDPE composites. By systematically varying the concentration, morphology, and size of fillers, as well as the non-bonded interaction strength expressed through different interatomic potentials, we have identified the contrasting roles of spherical fullerene and layered graphene fillers. C_60_, C_180_, and C_540_ fullerenes induce nearly equivalent enhancement of the composite elastic modulus at given filler concentration to within statistical error. In contrast, stiffness enhancement in composites with graphene flakes exhibits strong filler-size dependence. At very small flake sizes (C_61_, C_181_), comparable to those of typical fullerenes, there is little to no improvement in the stiffness of the composite with increasing graphene filler concentration. With increasing flake size, however, we have found an appreciable improvement in the composite modulus, with the effect of using C_541_ flakes being comparable to those of the fullerenes and with larger flakes (C_1087_, C_2161_) outperforming the fullerenes as fillers. This composite stiffness enhancement likely arises from more efficient load-transfer via interfacial shear (shear-lag) as well as ordering and densification of the polymer chains at the larger planar surfaces provided by larger graphene flakes. Within the limited statistics of graphene flake sizes considered in this study, the increase in composite modulus scales linearly with the flake size (circular flake radius). Given that typical solution-processed graphene flakes are much larger^4^ than the largest flakes studied here, we expect a sustained improvement in the composite stiffness with increasing flake size, a typical flake size for optimal reinforcement being in the 1–10 μm range^36^. It is also worth noting that a very recent experimental study of poly(vinyl acetate)/silica in the glassy phase has provided direct confirmation of an interfacial layer at the filler–matrix interface that consists of stretched polymer chains with an intrinsically higher interfacial elastic modulus than the matrix^37^. That work also demonstrates that the experimentally observed composite stiffness enhancement is best captured by a continuum model that accounts explicitly for the mechanical properties of the interfacial region (i.e., a three-phase model consisting of filler, interface, and matrix) rather than conventional two-phase composite models, which adds further credence to our observation in this work that the details of the filler–matrix interface are important beyond considerations of load transfer alone.

Studies of HDPE composites with larger graphene fillers are underway with the aim of establishing scaling laws for modulus enhancement over a couple of orders of magnitude of flake size as well as elucidating the chain dynamics and organization at the graphene–PE interface. Studies of nonlinear deformation and failure of fullerene- and graphene-reinforced HDPE composites also are underway and will be reported in future publications. Finally, in the present study, we have focused on the simplest model for matrix–filler interactions governed by weak dispersive forces. To the extent that strong covalent bonding between the filler and matrix is not significant, we expect that the qualitative behavior of polymer densification at the graphene–polymer interface as well as the quantitative power-law scaling relationship for composite stiffness should hold for other modes of filler–matrix interactions including, for example, hydrogen bonding between graphene oxide flakes and the polymer matrix. The use of functionalized graphene fillers could of course result in other modes of chemical bonding with the polymer matrix, and the effects of such stronger bonding of the composite’s constituents on its mechanical properties will be explored elsewhere.

## Additional Information

**How to cite this article**: Lu, C.-T. *et al*. A Comparison of the Elastic Properties of Graphene- and Fullerene-Reinforced Polymer Composites: The Role of Filler Morphology and Size. *Sci. Rep.* **6**, 31735; doi: 10.1038/srep31735 (2016).

## Supplementary Material

Supplementary Information

## References

[b1] Geim A. K. & Novoselov K. S. The rise of graphene. Nat. Mater. 6, 183–191 (2007). 17330084 10.1038/nmat1849

[b2] Castro Neto A. H., Guinea F., Peres N. M. R., Novoselov K. S. & Geim A. K. The electronic properties of graphene. Rev. Mod. Phys. 81, 109–162 (2009).

[b3] Dreyer D. R., Ruoff R. S. & Bielawski C. W. From concept to realization: an historical account of graphene and some perspectives for its future. Angew. Chem. Int. Ed. 49, 9936–9344 (2010). 10.1002/anie.201003024 21110353

[b4] Stankovich S. . Graphene-based composite materials. Nature 442, 282–286 (2006). 16855586 10.1038/nature04969

[b5] Kim H., Abdalla A. A. & Macosko C. W. Graphene/Polymer nanocomposites. Macromolecules 43, 6515–6530 (2010).

[b6] Potts J. R., Dreyer D. R., Bielawski C. W. & Ruoff R. S. Graphene-based polymer nanocomposites. Polymer 52, 5–25 (2011).

[b7] Cai D. & Song M. Recent advance in functionalized graphene/polymer nanocomposites. J. Mater. Chem. 20, 7906–7915 (2010).

[b8] Ramanathan T. . Functionalized graphene sheets for polymer nanocomposites. Nature Nanotech. 3, 327–331 (2008). 10.1038/nnano.2008.96 18654541

[b9] Li Y., Kroger M. & Liu W. K. Nanoparticle geometrical effect on structure, dynamics and anisotropic viscosity of polyethylene nanocomposite. Macromolecules. 45, 2099–2112 (2012).

[b10] Rissanou A. N. & Harmandaris V. A molecular dynamics study of polymer/graphene nanocomposites. Macromol. Symp. 331-332, 43–49 (2013).

[b11] Rissanou A. N., Power A. J. & Harmandaris V. Structural and dynamical properties of polyethylene/graphene nanocomposites through molecular dynamics simulations. Polymers 7, 380–417 (2015).

[b12] Rissanou A. N. & Harmandaris V. Dynamics of various polymer/graphene interfacial systems through atomistic molecular dynamics simulations. Soft Matter 10, 2876–2888 (2014). 24667937 10.1039/c3sm52688g

[b13] Yang H., Zhao X. J., Lu Z. Y. & Yan F. D. Temperature influence on the crystallization of polyethylene/fullerene nanocomposites: molecular dynamics simulation. J. Chem. Phys. 131, 234906 (2009). 20025347 10.1063/1.3275003

[b14] Adnan A., Sun C. T. & Mahfuz H. A molecular dynamics simulation study to investigate the effect of filler size on elastic properties of polymer nanocomposites. Compos. Sci. Technol. 67, 348–356 (2007).

[b15] Ferdous S. F., Sarker M. F. & Adnan A. Role of nanoparticle dispersion and filler-matrix interface on the matrix dominated failure of rigid C60-PE nanocomposites: a molecular dynamics simulation study. Polymer. 54, 2565–2576 (2013).

[b16] Zhang J. & Jiang D. Molecular dynamics simulation on mechanical performance of graphene/graphene oxide paper based polymer composites. Carbon. 67, 784–791 (2013).

[b17] Han Y. & Elliott J. Molecular Dynamics simulations of the elastic properties of polymer/carbon nanotube composites. Comput. Mater. Sci. 39, 315–323 (2007).

[b18] Frankland S. J. V., Harik V. M., Odegard G. M., Brenner D. W. & Gates T. S. The stress-strain behavior of polymer-nanotube composites from molecular dynamics simulation. Compos. Sci. Technol. 63, 1655–1661 (2003).

[b19] Griebel M. & Hamakers J. Molecular dynamics simulations of the elastic moduli of polymer–carbon nanotube composites. Comput. Methods Appl. Mech. Engrg. 193, 1773–1788 (2004).

[b20] Mayo S. L., Olafson B. D. & Goddard W. A. Dreiding: a generic force field for molecular simulations. J. Phys. Chem. 94, 8897–8909 (1990).

[b21] Capaldi F. M., Boyce M. C. & Rutledge G. C. Molecular response of a glassy polymer to active deformation. Polymer 45, 1391–1399 (2004).

[b22] Buell S., van Vliet K. J. & Rutledge G. C. Mechanical properties of glassy polyethylene nanofibers via molecular dynamics simulations. Macromolecules 42, 4887–4895 (2009).

[b23] Hossain D. . Molecular dynamics simulations of deformation mechanisms of amorphous polyethylene. Polymer 51, 6071–6083 (2010).

[b24] Cornell W. D. . A second generation force field for the simulation of proteins, nucleic acids, and organic molecules. J. Am. Chem. Soc. 117, 5179–5197 (1995).

[b25] Allen M. P. & Tildesley D. J. Computer Simulation of Liquids (Oxford: Clarendon, 1987).

[b26] Kong C. L. Combining rules for intermolecular potential parameters. II. Rules for the Lennard-Jones (12-6) potential and the Morse potential. J. Chem. Phys. 59, 2464 (1973).

[b27] Schwerdtfeger P., Wirz L. & Avery J. Program fullerene—a software package for constructing and analyzing structures of regular fullerenes. J. Comput. Chem. 34, 1508–1526 (2013). 23559399 10.1002/jcc.23278

[b28] Plimpton S. Fast parallel algorithms for short-range molecular dynamics. J. Comput. Phys. 117, 1–19 (1995).

[b29] Thijssen J. M. Computational Physics (New York: Cambridge, 1999).

[b30] Brandup J. & Immergut E. H. Polymer Handbook 3rd edn (New York: Wiley, 1989).

[b31] Dionne P. J., Ozisik R. & Picu R. C. Structure and dynamics of polyethylene nanocomposites. Macromolecules 38, 9351–9358 (2005).

[b32] Karayiannis N., Mavrantzas V. G. & Theodorou D. N. A novel Monte Carlo scheme for the rapid equilibration of atomistic model polymer systems of precisely defined molecular architecture. Phys. Rev. Lett. 88, 105503 (2002). 11909369 10.1103/PhysRevLett.88.105503

[b33] Bowden P. E. The elastic modulus of an amorphous glassy polymer. Polymer 9, 449–454 (1968).

[b34] Torquato S. Random Heterogeneous Materials: Microstructure and Macroscopic Properties (New York: Springer-Verlag, 2005).

[b35] Cox H. L. The elasticity and strength of paper and other fibrous materials. Brit. J. Appl. Phys. 3, 72–79 (1952).

[b36] Gong L. . Interfacial stress transfer in a graphene monolayer nanocomposite. Adv. Mater. 22, 2694–2697 (2010). 20473982 10.1002/adma.200904264

[b37] Cheng S. . Unraveling the Mechanism of Nanoscale Mechanical Reinforcement in Glassy Polymer Nanocomposites. Nano Lett. 16, 3630–3637 (2016). 27203453 10.1021/acs.nanolett.6b00766

