# Supplementary material for: A Comparison of the Elastic Properties of Graphene- and Fullerene-Reinforced Polymer Composites: The Role of Filler Morphology and Size
Source: Sci Rep. 2016 Aug 22;6:31735. doi: 10.1038/srep31735 (PMC4992834; doi:10.1038/srep31735)
Supplement: Supplementary Information [file srep31735-s1.pdf]

Supplementary Information

A Comparison of the Elastic Properties of Graphene- and Fullerene-Reinforced Polymer Composites: The Role of Filler Morphology and Size

Chang-Tsan Lu[1], **Asanka Weerasinghe**[2], **Dimitrios Maroudas**,[1,#] **and Ashwin Ramasubramaniam**[3,*]

[1] Department of Chemical Engineering, University of Massachusetts, Amherst, MA 01003, U.S.A.

[2] Department of Physics, University of Massachusetts, Amherst, MA 01003, U.S.A.

[3] Department of Mechanical and Industrial Engineering, University of Massachusetts, Amherst, MA 01003, U.S.A.

[#] maroudas@ecs.umass.edu

[*] ashwin@umass.edu

1. Interaction Parameters for Composites with Polyethylene Matrix and Carbon Fillers

The bonding energy contribution consists of three components, E_{bond}, E_{angle}, and $E_{dihedral}$, arising from changes in the bond length (r), bond angle (θ), and dihedral angle (ϕ), respectively, which are given by the expressions

$$\begin{aligned} E_{bond} &= k_b (r - r_0)^2, \\ E_{angle} &= k_a (\theta - \theta_0)^2, \\ E_{dihedral} &= \sum_{n=0}^{3} A_n \cos^n \phi. \end{aligned} \tag{S1}$$

Non-bonded interactions are assumed to follow a standard 12-6 Lennard-Jones form,

$$E_{non-bonded} = 4\varepsilon_{\alpha\beta}\left[\left(\frac{\sigma_{\alpha\beta}}{r_{\alpha\beta}}\right)^{12} - \left(\frac{\sigma_{\alpha\beta}}{r_{\alpha\beta}}\right)^{6}\right], \tag{S2}$$

where α and β denote the two interacting united atoms; atoms within a distance equal to four C-C bond lengths of each other in the same polymer chain do not participate in such non-bonded interactions. For the HDPE matrix, we adopt two such parameterizations developed by Buell *et al.* [1] and Capaldi *et al.* [2], and referred to as HDPE 1 and HDPE 2, respectively. These potentials were originally developed by Paul et al. [3] and modified subsequently as described in Refs. 1 and 2. The potentials describe with reasonable accuracy a broad range of static and dynamic properties of *n*-alkane melts including *P–V–T* behavior, x-ray scattering profiles, and self-diffusion behavior, among others. The relevant parameters in Equations (S1) and (S2) for these two potentials are listed in Table S1. Nanocarbon fillers are also described using a Dreiding potential [4]; the relevant parameters entering Equations (S1) and (S2) are listed in Table S2. Finally, filler–matrix interactions are described as non-bonded interactions using standard Lorentz-Berthelot mixing rules [5] resulting in the interaction parameters listed in Table S3.

Table S1: United atom parameters in Equations (1) and (2) for polyethylene (HDPE) based on the parameterizations by Buell *et al.* [1], HDPE 1, and Capaldi *et al*. [2], HDPE 2.

Parameter	HDPE 1 [1]	HDPE 2 [2]
k_b (kcal/mol/Å^2)	349.896	239.006
r_0 (Å)	1.53	1.53
k_a (kcal/mol/deg^2)	59.999	60.947
θ_0 (deg)	109.5	110.0
A_0 (kcal/mol)	1.562	1.73
A_1 (kcal/mol)	4.051	-4.493
A_2 (kcal/mol)	0.867	0.776
A_3 (kcal/mol)	-6.48	6.991
$\varepsilon_{CH2\text{-}CH2}$ (kcal/mol)	0.093	0.112
$\varepsilon_{CH2\text{-}CH3}$ (kcal/mol)	0.145	0.112
$\varepsilon_{CH3\text{-}CH3}$ (kcal/mol)	0.227	0.112
$\sigma_{CH2\text{-}CH2}$ (Å)	4.01	4.01
$\sigma_{CH2\text{-}CH3}$ (Å)	4.01	4.01
$\sigma_{CH3\text{-}CH3}$ (Å)	4.01	4.01

Table S2: United atom parameters in Equations (1) and (2) for carbon (C). [4]

**Parameter**	**C**
k_b (kcal/mol/Å^2)	469.0
r_0 (Å)	1.409
k_a (kcal/mol/deg^2)	63.0
θ_0 (deg)	120.0
A_0 (kcal/mol)	7.25
A_1 (kcal/mol)	0.0
A_2 (kcal/mol)	-7.25
A_3 (kcal/mol)	0.0
$\varepsilon_{\text{C-C}}$ (kcal/mol)	0.086
$\sigma_{\text{C-C}}$ (Å)	3.4

Table S3: Cross terms in Eq. (2) for non-bonded interactions in polyethylene–carbon composites.

**Parameter**	**HDPE 1/C**	**HDPE 2/C**
$\varepsilon_{\text{CH2-C}}$ (kcal/mol)	0.09	0.098
$\varepsilon_{\text{CH3-C}}$ (kcal/mol)	0.14	0.098
$\sigma_{\text{CH2-C}}$ (Å)	3.705	3.705
$\sigma_{\text{CH3-C}}$ (Å)	3.705	3.705

2. Structural, geometrical, and compositional details of simulated filler–matrix combinations

Nanocomposite models were prepared with the aim of gathering extensive statistics and identifying clear trends in the elastic response as a function of filler size, concentration, and geometry. Table S4 lists the various filler–matrix combinations used in this work; ten samples corresponding to each of these combinations were used in the mechanical tests to generate reliable statistics.

Table S4: Structural, geometrical, and compositional details of filler–matrix combinations considered in this work.

Filler type	Filler radius (Å)	Number of fillers	PE matrix	Filler weight ratio (%)
Fullerene C_{60}	3.54	9, 18, 27, 36, 45, 54, 63	200×100-mer	2.26, 4.42, 6.48, 8.46, 10.36, 12.18, 13.92
Fullerene C_{180}	5.98	3, 6, 9, 12, 15, 18, 21	200×100-mer	2.26, 4.42, 6.48, 8.46, 10.36, 12.18, 13.92
Fullerene C_{540}	10.36	1, 2, 3, 4, 5, 6, 7	200×100-mer	2.26, 4.42, 6.48, 8.46, 10.36, 12.18, 13.92
Graphene C_{61}	7.10	9, 18, 27, 36, 45, 54, 63	200×100-mer	2.30, 4.49, 6.58, 8.59, 10.51, 12.36, 14.32
Graphene C_{181}	12.30	3, 6, 9, 12, 15, 18, 21	200×100-mer	2.27, 4.44, 6.52, 8.51, 10.41, 12.24, 13.99
Graphene C_{541}	21.21	1, 2, 3, 4, 5, 6, 7	200×100-mer	2.26, 4.43, 6.50, 8.48, 10.38, 12.20, 13.95
Graphene C_{1087}	29.29	1, 2, 3, 4, 5, 6, 7	400×100-mer	2.27, 4.45, 6.52, 8.51, 10.42, 12.25, 14.00
Graphene C_{2161}	42.41	1, 2, 3	400×100-mer	4.42, 8.47, 12.19

3. Estimation of glass-transition temperature

Figure S1 displays representative density versus temperature curves that are generated over the (NPT) quenching process for neat as well as nanocarbon-reinforced HDPE. From these calibration curves, one can estimate the glass transition temperature (T_g) as indicated by the example constructions in Fig. S1. For neat HDPE—considering both parameterizations of Table S1—we estimated T_g to lie over the range 240-265 K, which contains the typical experimentally reported value of 250 K [6]; the addition of fillers results in a slight elevation of T_g to the range of 260-295 K.

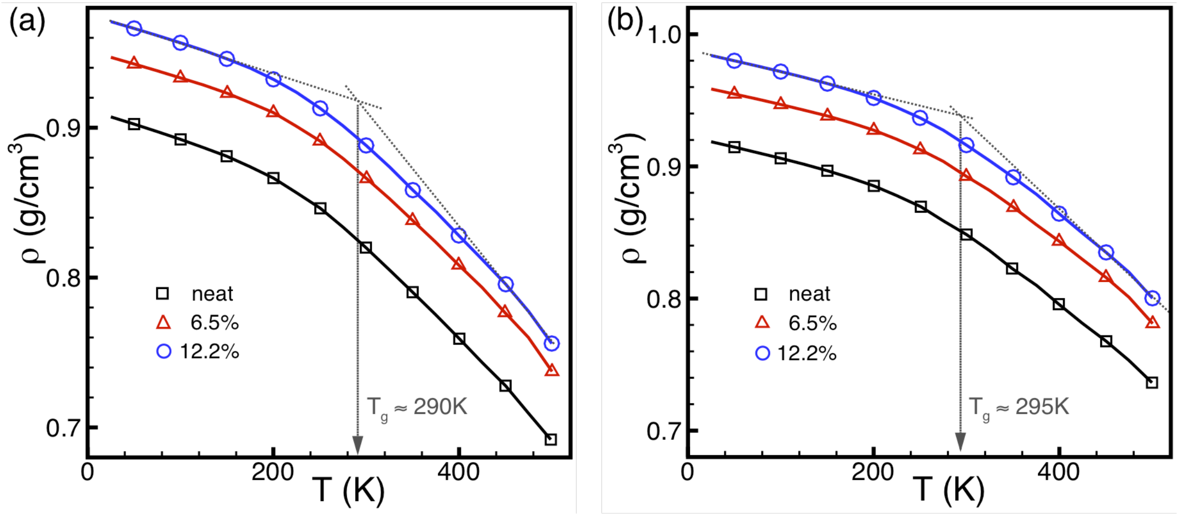

Figure S1: Representative plots of density vs. temperature for neat (squares), fullerene-reinforced (triangles), and graphene-reinforced (circles) HDPE from MD simulations using (a) HDPE1/C and (b) HDPE2/C parameters (see Tables S1-S3). Results for fullerene (C_{540}) and graphene (C_{1087}) filler concentrations (wt. %) are indicated. An example geometric construction for estimating the glass transition temperature (T_g) is indicated in each case. For all cases examined, T_g lies within the range of 240-300 K.

REFERENCES

1. Buell S., van Vliet K. J., Rutledge G. C., Mechanical properties of glassy polyethylene nanofibers via molecular dynamics simulations. *Macromolecules* **42**, 4887-4895 (2009).

2. Capaldi F. M., Boyce M. C., Rutledge G. C., Molecular response of a glassy polymer to active deformation. *Polymer* **45**, 1391-1399 (2004).

3. Paul W., Yoon D. Y., Smith G. D. An optimized united atom model for simulations of polymethylene melts. *J. Chem. Phys.* **103**, 1702–1709 (1995).

4. Cornell W. D., et al. A second generation force field for the simulation of proteins, nucleic acids, and organic molecules. *J. Am. Chem. Soc.* **117**, 5179–5197 (1995).

5. Allen M. P., Tildesley D. J. *Computer Simulation of Liquids* (Oxford: Clarendon, 1987).

6. Brandup J., Immergut E. H. *Polymer Handbook*. 3rd ed. (New York: Wiley, 1989).
